# The interplay between habitat structure and chemical contaminants on biotic responses of benthic organisms

**DOI:** 10.7717/peerj.1985

**Published:** 2016-05-03

**Authors:** Mariana Mayer-Pinto, Miguel G. Matias, Ross A. Coleman

**Affiliations:** 1 Centre for Research on Ecological Impacts of Coastal Cities, Marine Ecology Laboratories A11, School of Biological Sciences, The University of Sydney, NSW, Australia; 2 InBio/CIBIO, University of Évora, Largo dos Colegiais, Évora, Portugal; 3 Imperial College London, Silwood Park Campus, Ascot, Berkshire, United Kingdom

**Keywords:** Chemical disturbance, Pollution, Artificial turfs, Habitat complexity

## Abstract

Habitat structure influences the diversity and distribution of organisms, potentially affecting their response to disturbances by either affecting their ‘susceptibility’ or through the provision of resources that can mitigate impacts of disturbances. Chemical disturbances due to contamination are associated with decreases in diversity and functioning of systems and are also likely to increase due to coastal urbanisation. Understanding how habitat structure interacts with contaminants is essential to predict and therefore manage such effects, minimising their consequences to marine systems. Here, we manipulated two structurally different habitats and exposed them to different types of contaminants. The effects of contamination and habitat structure interacted, affecting species richness. More complex experimental habitats were colonized by a greater diversity of organisms than the less complex habitats. These differences disappeared, however, when habitats were exposed to contaminants, suggesting that contaminants can override effects of habitats structure at small spatial scales. These results provide insight into the complex ways that habitat structure and contamination interact and the need to incorporate evidence of biotic responses from individual disturbances to multiple stressors. Such effects need to be taken into account when designing and planning management and conservation strategies to natural systems.

## Introduction

The structural complexity of habitats has a profound influence on the distribution and abundance of organisms (e.g., Heck, 1979; Loke & Todd, 2016; MacArthur & MacArthur, 1961; McCoy & Bell, 1991) and functioning of systems (Graham & Nash, 2013), affecting ecological processes at all levels of organisation (Brown, 2007). Habitat structure influences, among other factors; the availability of resources, niche partitioning (Levins, 1979), competitive interactions (Jones, 1988) and the abundance of refuge from predators (Crowder & Cooper, 1982). Habitat complexity is usually positively associated with number of species (Kovalenko, Thomaz & Warfe, 2012), but this relationship can vary depending on the species or functional groups involved (Lassau et al., 2005; Scharf, Manderson & Fabrizio, 2006). Given the strong linkages between structure and ecological processes, it is likely that the structure of habitats can affect the response of organisms to disturbances (e.g., Gosper et al., 2015; Lindsay & Cunningham, 2009), changing the capability of assemblages to resist different types of impacts by either affecting their ‘susceptibility’, e.g., influencing the success of their predatory strategies (e.g., Karkarey et al., 2014), or through the provision of resources that can mitigate the impacts of disturbances (e.g., shelter and food; Caley & StJohn, 1996; Syms & Jones, 2000). Complex habitats can also facilitate recovery (e.g., Kovalenko, Thomaz & Warfe, 2012). In addition, because the structure of habitats influences composition of species (e.g., Matias, Underwood & Coleman, 2007), complex habitats can support communities that are probably more likely to include species that are tolerant to particular types of disturbances than less structured habitats.

Contamination by chemicals is a particular pervasive type of disturbance and is likely to increase due to, among other things, the increasing urbanisation of systems—usually derived from a greater influx of chemicals into natural systems through discharges of stormwater and sewage and industrial and agricultural practices (Rohr, Kerby & Sih, 2006; Schiedek et al., 2007). In fact, contaminants are present in most systems worldwide and are considered one of the largest threats to many aquatic species (Rohr, Kerby & Sih, 2006), being associated with a global decrease in species richness and changes in functioning (Johnston, Mayer-Pinto & Crowe, 2015; Johnston & Roberts, 2009). It is believed, however, that impacts due to contamination can be predicted, and therefore managed, considering (i) the type of toxicants (and their chemical properties), (ii) the functional groups present in the impacted area, (iii) their reproductive rates, (iv) the trophic interactions occurring in the systems and the (v) functions that these organisms (or functional groups) perform in these systems (Halstead et al., 2014). We consider, however, that knowledge on interactions of contaminants with the structure of habitats is also essential to increase our ability to predict not only biotic responses to contaminants, but also to elucidate some of the mechanisms that habitat structure influence diversity.

Abiotic factors can mediate or influence the strength and/or dynamics of biological interactions, in terrestrial and marine systems, in various and complex ways. For instance, increases in structural complexity at the habitat level may increase the abundance of invertebrate predators and parasitoids (Langellotto & Denno, 2004). At the same time, contaminants, such as pesticides, can alter competitive interactions and/or predator–prey interactions by favouring competitively inferior species or by having strong asymmetric effects on communities, e.g., herbicides often have negative effects on herbivores by reducing plant availability (Fleeger, Carman & Nisbet, 2003; Rohr & Crumrine, 2005; Rohr, Kerby & Sih, 2006).

Here we tested the hypothesis that effects of different chemical contaminants interact differently with habitat structure influencing the colonization of benthic habitats. We addressed this question using manipulative experiments done in the field including two types of artificial mimics of coralline turfs as experimental habitats and three types of contaminants as model agents of chemical disturbances.

## Material & Methods

### Experimental design

The experiment was done in each of two sites on a moderately exposed rocky shore in the Cape Banks Scientific Marine Research Area, hereafter referred as Cape Banks, in Botany Bay, Australia (33.59°S; 151.14°E; NSW Fisheries Permit F96/146). Sites were chosen amongst meadows of algal turf dominated by *Corallina officinalis* L and were approximately 200 m apart. Experimental habitats made of synthetic turfs (15 × 15 cm) were used to mimic the structure of natural coralline turfs (Kelaher, 2002; Matias, Underwood & Coleman, 2007). These artificial habitats are colonised by diverse assemblages of polychaetes, amphipods and molluscs from a range of classes, families, feeding modes, mobility, etc. (Beesley, Ross & Wells, 1998; Matias et al., 2010). Most of these organisms are quite small, ranging from 0.5 to 3 mm in size (Matias, Underwood & Coleman, 2007; Matias et al., 2010) and the width of artificial habitats is more than 200 times their average body lengths (i.e., <1 mm; Matias et al., 2010). Experimental habitats were interspersed on rock-platforms or large boulders, between 0.3 and 0.6 m above mean low water (MLW) and attached to shore using stainless-steel screws and rubber washers. The experimental habitats were deployed for six weeks, from October 2009 to November ’09, which has been shown to be enough time for colonizing assemblages to differ between habitats with different structural complexities (Matias, Underwood & Coleman, 2007; Matias et al., 2010). This time period was chosen as a compromise between allowing enough time for colonization and ensuring the safe recovery of experimental units. We are confident that six weeks were enough to allow colonization representative assemblages since previous studies have shown artificial turfs are rapidly colonized after only a few days (e.g., six days), achieving large numbers of individuals after only month (Olabarria, 2002). Experimental habitats were made of two different types of synthetic turfs (Grassman Pty Ltd., NSW, Australia): *short* (i.e., 10 mm; 66.2 number of fronds per cm^2^) and *long* (i.e., 40 mm; 16.2 number fronds per cm^2^) turfs. These synthetic turfs were chosen because of their difference in length and in density of fronds, thus maximizing the structural differences needed to test our hypotheses about different types of habitats. Previous studies have consistently shown significant differences in numbers of species between the *short* and *long* turfs (Matias et al., 2010; Matias et al., 2011).

The patches of artificial turfs were attached with cable-ties to two overlapping layers (of 35 × 35 cm) of plastic coated green wire mesh (12.7 mm aperture), which prevented the patches from breaking and being washed away in the field (see Matias, Underwood & Coleman, 2007). A plastic container of 12.5 cm of diameter and 3 cm height were placed directly underneath the patch and between the two layers of mesh and another piece of mesh (35 cm × 35 cm). The containers were affixed with cable ties to ensure they would not be displaced due to waves. Plaster blocks with or without contaminants were placed inside the containers. Plaster blocks have been used to deliver contaminants to assemblages in experimental tests of hypotheses about pollution because they release contaminants slowly and continuously into sediments (e.g., Lindegarth & Underwood, 1999; Morrisey, Underwood & Howitt, 1996) and on hard substrata (e.g., Johnston & Keough, 2000; Mayer-Pinto et al., 2011). Control treatments had no plaster blocks, to evaluate any possible effects of the plaster itself (Cartwright, Coleman & Browne, 2006). Prior to installation, 20 holes of 4 mm of diameter were drilled in all patches of artificial turf to allow the release of contaminants through the patches and to prevent the contaminants from being washed away too rapidly (see Mayer-Pinto et al., 2011). Three replicates of each treatment at each site were analysed. After 6 weeks, each patch was washed in a 500 µm sieve and all invertebrates sorted and counted under a binocular microscope at 16× magnification. All molluscs were identified to the finest possible taxonomic resolution, either species or morphospecies.

### Contaminants dosing

Plaster blocks were made of 1,800 g of dental plaster and 1,050 ml of water. In order to deliver contaminants to assemblages, carbaryl, iron phosphate or metaldehyde were added to plaster blocks. These contaminants, used as model agents of chemical disturbances, i.e., pesticide and metals, are commonly found in coastal and estuarine areas (e.g., McCready, Birch & Long, 2006), and influence different components of the assemblages. Carbaryl is among the top 10 pesticides used in the United States and can be applied directly onto aquatic habitats or can end up in these habitats via unintentional overspray, aerial drift or runoff (Relyea, 2005). Metaldehyde is considered ‘an emergent contaminant’ and high levels of this chemical has been detected in surface waters (Moreau, Burgeot & Renault, 2015). The doses applied here were the same as used in previous studies that had shown effects of these contaminants on at least one taxonomic group (Poore, Campbell & Steinberg, 2009; Rae, Robertson & Wilson, 2009; Speiser & Kistler, 2002). Carbaryl decreased numbers of arthropods, mainly amphipods, (Poore, Campbell & Steinberg, 2009) whereas iron phosphate and metaldehyde influenced numbers of terrestrial molluscs (Rae, Robertson & Wilson, 2009; Speiser & Kistler, 2002). Blank plaster blocks were made without the addition of any contaminant. Plaster blocks were contaminated with 189 g of wettable powder carbaryl (80% carbaryl) resulting in 7.6% carbaryl by weight (as described by Poore, Campbell & Steinberg, 2009). Blocks contaminated with iron phosphate had 250 g of contaminant, resulting in 5% iron phosphate by weight. We used Defender^®^ (15 g of metaldehyde per kg) snail and slug pellets as the third contaminant. 33.5 g of pellets were dissolved in water and then mixed with plaster giving a dose of 1.5% metaldehyde by weight. These concentrations were used as per Speiser & Kistler (2002) and Rae, Robertson & Wilson (2009). All contaminants were dissolved into water before being mixed with the plaster. Plaster blocks were changed every two weeks. Approximately 200 ml of plaster blocks (with or without contaminants) were moulded in the containers (see design in Supplemental Information).

**Table 1:** Analyses of variance of mean total number of species in each treatment. Site (Si) was random with 2 levels. Habitat type (Ha) and Contaminant (Contam) were fixed and orthogonal, with 2 and 5 levels, respectively; *n* = 3. Data were pooled when *p* > 0.25.

Source	df	MS	Pseudo-*F*
Site (Si)	1	1148.1	23.93^**^
Habitat type (Ha)	1	631.73	13.17^**^
Contaminant (Contam)	4		
C1—Control vs Carbaryl	1	155.04	3.24^ns^
C2—Control vs Iron	1	4.17E–02	0.00^ns^
C3—Control vs Metal	1	63.375	1.48^ns^
C4—Control vs Blank	1	12.443	0.28^ns^
Ha × Contam	4		
Ha × C1	1	301.04	6.29^*^
Ha × C2	1	210.04	3.87^ns^
Ha × C3	1	273.38	6.38^*^
Ha × C4	1	68.387	1.52^ns^
Pooled	49	47.977	
Total	59		
Pair-wise tests	Ha × C1—Control—Short < Long Carbaryl—Short = Long Ha × C3—Control – Short < Long Metal–Short = Long Short turfs – Carbaryl = Control Metal = Control Long turfs—Carbaryl < Control Metal < Control		

**Notes.**

ns, not significant (*p* > 0.05).

* *p* < 0.05.

** *p* < 0.01.

#### Statistical analyses

Analyses of variance were used to test whether there were differences in the total number of species, abundance of the most common taxonomic groups and structure of assemblages between treatments (detailed in Tables). *A priori* contrasts were done to compare differences between contaminants and controls, since we did not have specific hypotheses about the magnitude of effects among contaminants (e.g., Carbaryl vs. Control; Iron vs. Controls, etc.). Appropriate *F*-ratios were constructed with Mean Squares (MS) calculated in the PERMANOVA add-on for PRIMER 6 using a similarity matrix based on Euclidean distances (Anderson, Gorley & Clarke, 2007). PERMANOVA *F*-ratios for univariate analyses using Euclidean distances are analogous to ANOVA Fisher’s *F* statistic, which has a known distribution under a true null hypothesis (Anderson, Gorley & Clarke, 2007). The *F* distribution was used to obtain *p* values. Prior to all analyses, the assumption of homogeneity of variance was examined using Cochran’s C test. When Cochran’s test was significant, data were transformed. Factors in the ANOVA were pooled when *p* > 0.25 to increase the power of tests above the pooled term (see Underwood, 1997). To determine whether there were significant differences between treatments regarding relative abundance and composition of assemblages within each treatment, multivariate analyses were done using PERMANOVA in PRIMER 6 (Anderson, Gorley & Clarke, 2007). There were no effects of plaster on the structure and composition of assemblages (PERMANOVA; *p* > 0.05) or on the total number of species within patches (Tables 1 and 2). Therefore, comparisons were done with the patches without plaster blocks).

**Table 2:** Analyses of variance of the number of species and abundance of gastropods in each treatment. Site (Si) was random with 2 levels. Habitat type (Ha) and Contaminant (Contam) were fixed and orthogonal, with 2 and 5 levels, respectively; *n* = 3. Data were pooled when *p* > 0.25.

Source	df	Number of species		Abundance	
		MS	Pseudo-*F*	MS	Pseudo-*F*
Site (Si)	1	160.8	10.62^**^	21.2	1.25^ns^
Habitat type (Ha)	1	240.9	15.92^**^	5.3	0.31^ns^
Contaminant (Contam)	4				
C1—Control vs Carbaryl	1	37.5	2.39^ns^	13.3	0.88^ns^
C2—Control vs Iron	1	0.0	0.00^ns^	0.0	0.00^ns^
C3—Control vs Metal	1	16.7	1.43^ns^	0.0	0.00^ns^
C4—Control vs Blank	1	4.4	0.35^ns^	5.0	0.24^ns^
Ha × Contam	4				
Ha × C1	1	80.7	5.14^*^	78.5	5.22^*^
Ha × C2	1	40.0	2.33^ns^	24.7	1.78^ns^
Ha × C3	1	73.5	6.32^*^	96.4	6.30^*^
Ha × C4	1	16.0	1.26^ns^	123.8	6.12^*^
Pooled	49	15.1		17.1	
Total	59				
Pair-wise tests	Ha × C1 – Control – Short < Long Carbaryl – Short = Long Ha × C3—Control – Short < Long Carbaryl—Short = Long Short turfs —Carbaryl = Control Long turfs – Carbaryl < Control		Ha × C1 – Control – Short < Long Carbaryl – Short = Long Ha × C3 - Control – Short < Long Carbaryl – Short = Long Short turfs – Carbaryl = Control Long turfs – Carbaryl < Control	

**Notes.**

ns, not significant (*p* > 0.05).

* *p* < 0.05.

** *p* < 0.01.

Data were log(*x* + 1); Cochran’s test *p* < 0.05.

Analyses were run using two different similarity matrices: Bray–Curtis on untransformed data and Jaccard dissimilarities. When run on untransformed data, Bray–Curtis gives more weight to changes in species abundances, whereas Jaccard is based on changes in species composition (e.g., presence-absences) and does not take into account changes in species relative abundances (Clarke & Warwick, 2001). When used in combination, these two measures of similarity allow assessing the relative importance of changes in species abundances or composition. For all analysis, we used 9,999 permutations under a reduced model (Anderson, Gorley & Clarke, 2007).

We used a random sampling (or bootstrapping) procedure to assess whether differences in the total number of species between long and short turfs changed depending on the presence of a particular contaminant. The random sampling procedure consists in calculating the log ratio between of number of species in long turfs by the numbers of species in short turfs ($\log ({S}_{\mathrm{long}}/{S}_{\mathrm{short}})$) for all possible pairs of replicates. This procedure allows us to generate a mean and standard deviation of the comparison between long and short turfs.

## Results

### Total number of species and taxonomic groups

**Figure 1:**
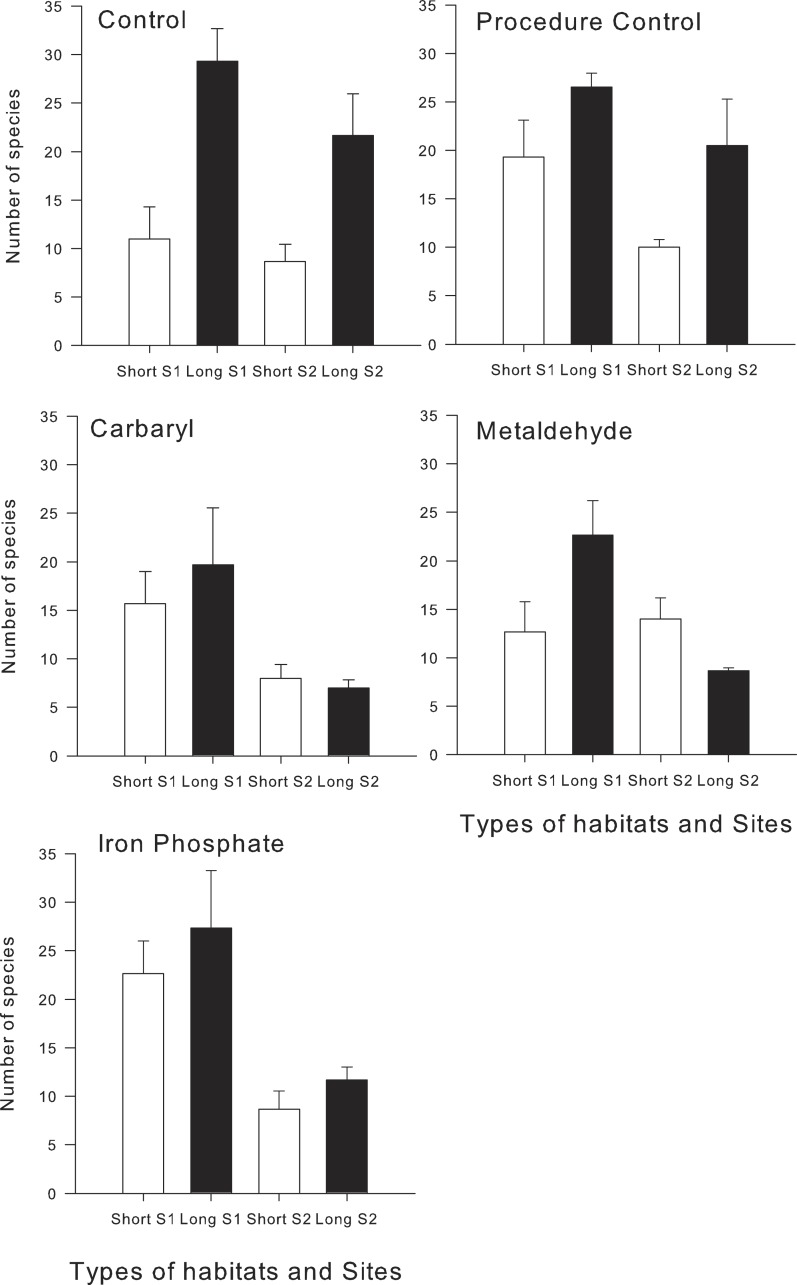
Mean (±SE) of the total number of species in each treatment and type of experimental habitat. White bars indicate short turfs and black bars indicate long type of turfs.

Analysis of numbers of species revealed the importance of the interplay between habitat structure and chemical contaminants. There were significant interactions between habitat type and the contaminants carbaryl and metaldehyde on the total number of species (Table 1). In the control synthetic turfs (hereafter controls), a greater number of species colonised the long type of turf than the shorter one, whereas on the treatments exposed to carbaryl and metaldehyde, there were no significant differences between habitats (Pair-wise test; *p* > 0.05; Table 1 and Fig. 1). There were no significant effects of iron on overall number of species in the two types of habitats (i.e., long and short; pseudo-*F*_1,49_ = 3.87; *p* > 0.05; Table 1), although the contaminant did reduce the difference between types of habitats shown in the control treatments (Fig. 1).

**Figure 2:**
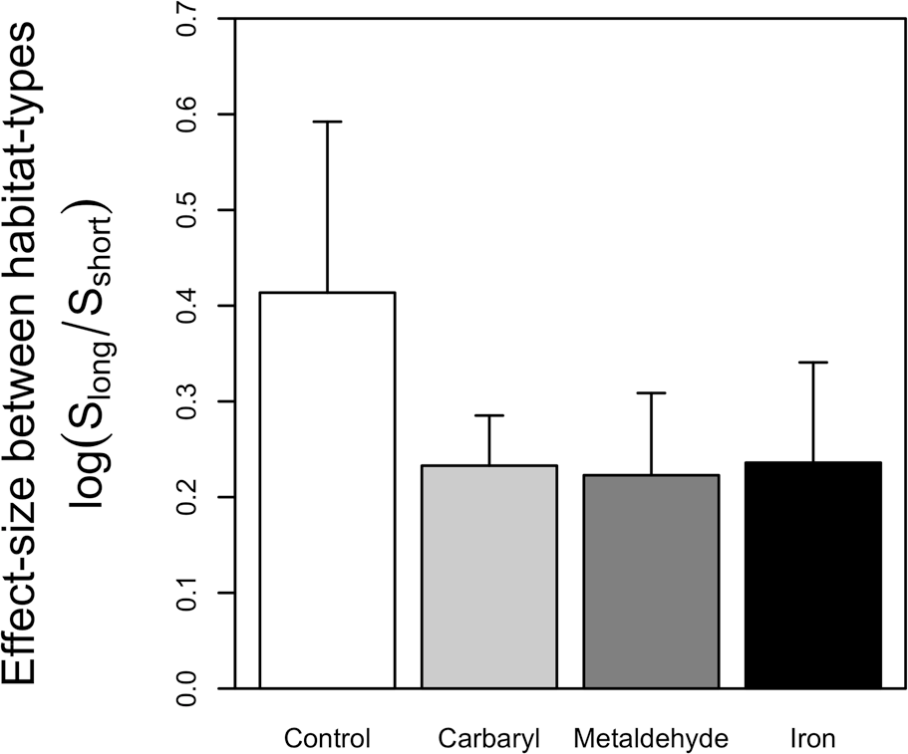
Graph indicating the effect-size between habitats-types in the controls and when each type of contaminant was added. Greater effect-sizes indicate greater differences in numbers of species between longer and short habitats. Errors bars indicate standard deviation calculated across all possible pairs of replicates for each combination of contaminant and type of turf.

The random sampling procedure clearly showed that effects of contaminants mediate effects of habitat structure, significantly decreasing the difference in the number of species between habitats with different complexities (Fig. 2). This was mainly due to a significant reduction in the number of species in the long turfs caused by the contaminants (Table 1 and Fig. 1).

**Figure 3:**
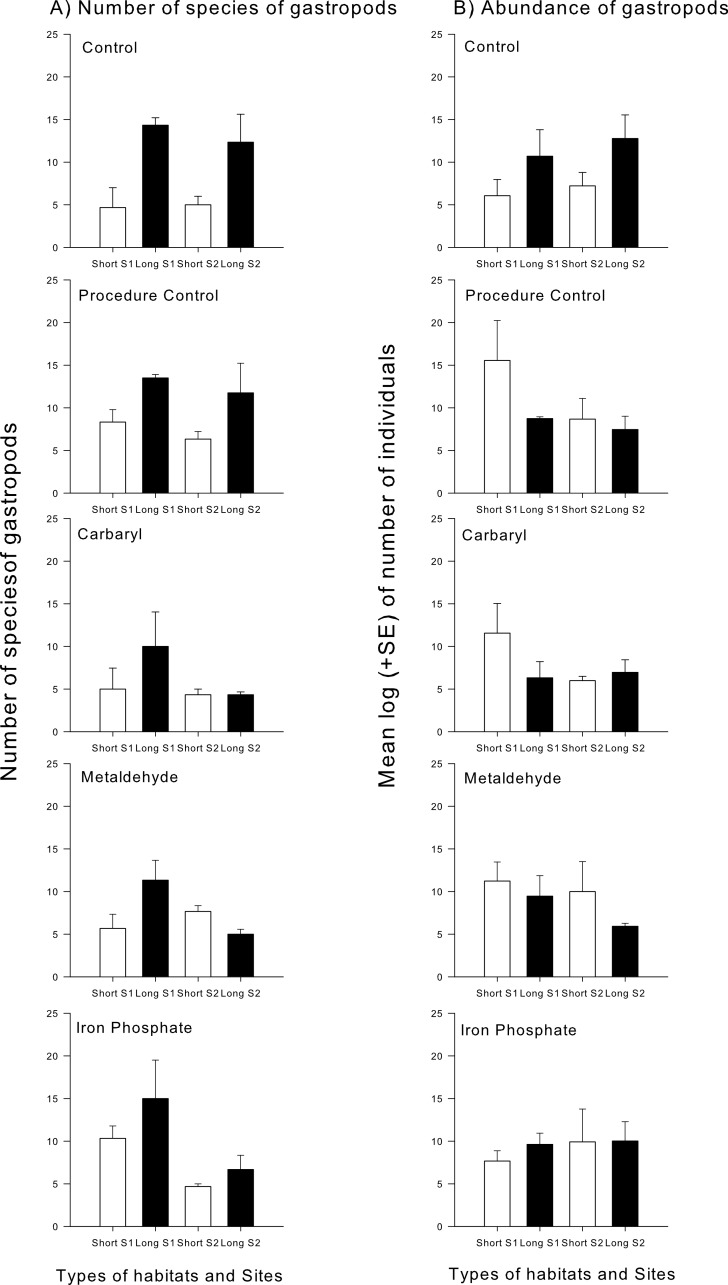
Mean (+SE) number of species (A) and mean log (±SE) of the number of individuals’ gastropods (B). White bars indicate short turfs and black bars indicate long type of turfs.

**Figure 4:**
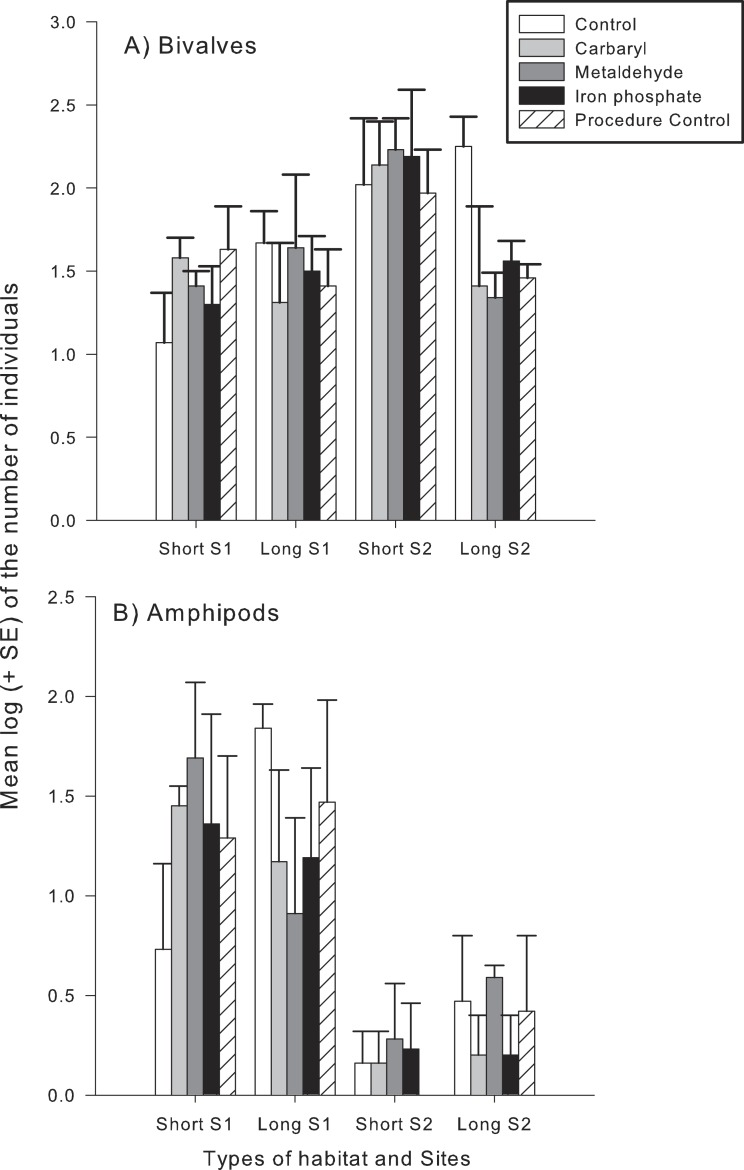
Mean log (+SE) of the number of individuals of (A), bivalves (B) and amphipods in each type of experimental habitat and each contaminant treatment. Bars with different colours indicate different treatments: controls (white), Carbaryl (light-gray), Metaldehyde (dark-gray), Iron (black) and Procedural controls (striped).

Different taxonomical groups showed divergent responses to contaminants. There was a significant interaction in the number of species and abundance of gastropods between habitat type and the contaminants carbaryl and metaldehyde. A greater number of individuals and species of gastropods colonised the long type of synthetic turfs than the short turfs, but only in the control treatments (i.e., no contaminants). When in the presence of carbaryl or metaldehyde, there were no significant differences between habitat types (Table 2 and Fig. 3). There was, however, an effect of the plaster on the abundance of gastropods (Pseudo-*F*_1,49_ = 6.12; *p* < 0.05), so differences found between treatments might be due to an artefact effect of the procedure used and should be interpreted with care. There also was a significant interaction between habitat type and the contaminant carbaryl regarding the abundance of amphipods and bivalves, but the *a posteriori* tests could not identify where these differences occurred (Table 3; SNK; *p* > 0.05). The graphs indicate however a similar pattern found in the previous results, i.e., greater abundance on the long turf than on the short turf, but only at the control treatments (Fig. 4). It was not possible to analyse differences regarding number of species within these groups due to inconsistent taxonomic resolution. Iron did not have an effect on any particular taxonomic group (Tables 2 and 3).

### Assemblages

**Figure 5:**
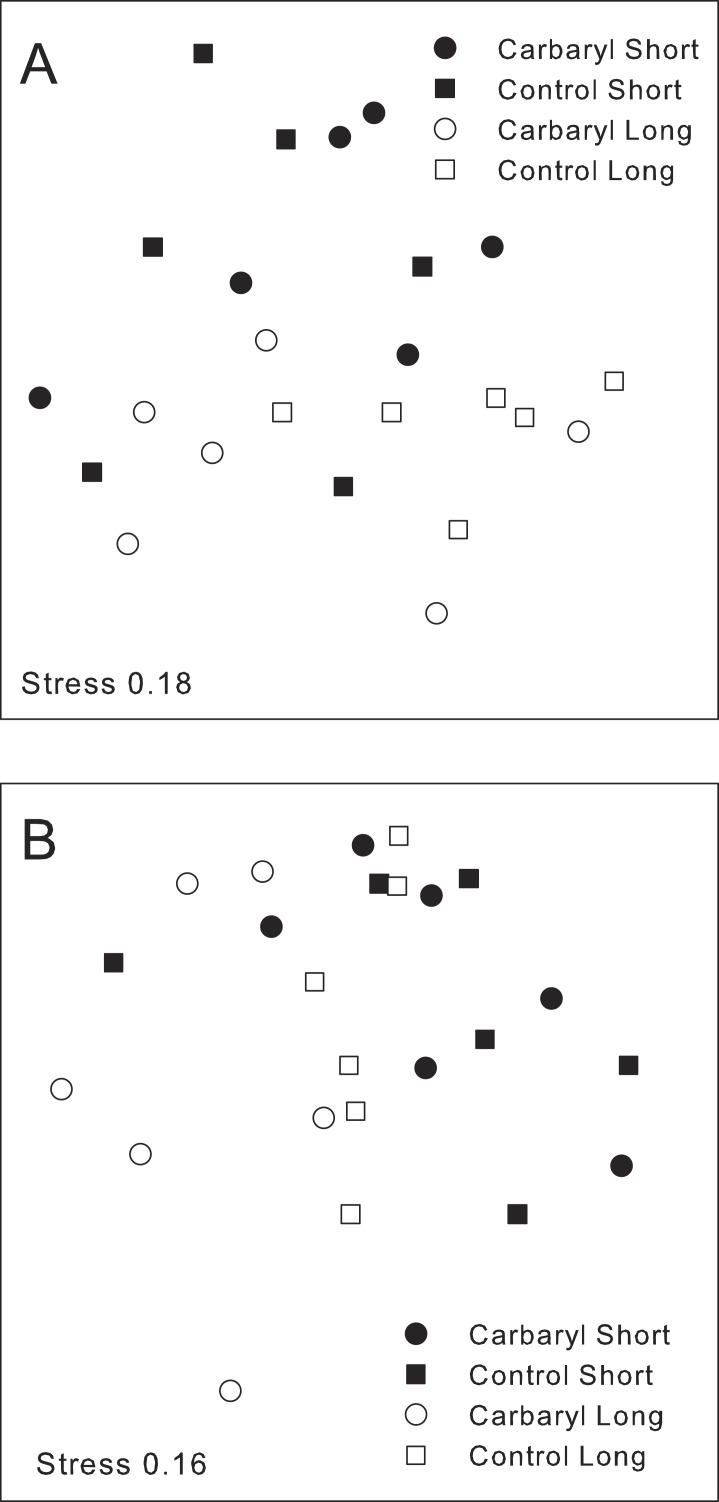
nMDS, done with the Jaccard index (A) and Bray-Curtis index (B), of colonising assemblages in the short and long turfs exposed to carbaryl and control treatments at Cape Banks.

Analysis of entire assemblages did not reveal major effects of contaminants, regardless of habitat type. The structure and composition of the colonising assemblages varied with habitat type (i.e., short vs long turfs) and sites (Table 4). There were, however, no significant differences in the whole structure of assemblages (Bray–Curtis index) exposed to different types of contaminants. Nevertheless, the composition of species (Jaccard index) differed between controls and habitats exposed to carbaryl, only in longer turfs (pseudo-*F*_1,49_ = 1.82; *p* < 0.05; Table 4 and Fig. 5).

## Discussion

Our results showed that effects of contamination and habitat structure interact, affecting species richness. More complex experimental habitats (i.e., long artificial turfs) were colonized by a greater diversity of organisms than the less complex habitats (short artificial turfs), which is consistent with results from previous studies (e.g., Matias, Underwood & Coleman, 2007; Matias, Underwood & Coleman, 2010; Matias et al., 2010). This difference disappeared, however, when experimental habitats were exposed to particular types of contaminants (i.e., carbaryl and metaldehyde), mainly due to a reduction of number of species on long turfs caused by the chemicals. These results are consistent with the model that chemical disturbances mediate effects of habitat structure. Effects of contaminants can therefore override the important role of habitat heterogeneity in supporting species diversity in small spatial scales.

**Table 3:** Analyses of variance of some of the most abundant taxonomic groups in each treatment. Site (Si) was random with 2 levels. Habitat type (Ha) and Contaminant (Contam) were fixed and orthogonal, with 2 and 5 levels, respectively; *n* = 3. Data were pooled when *p* > 0.25.

Source	df	Bivalves^t^		Amphipods	
		MS	Pseudo-*F*	MS	Pseudo-*F*
Site (Si)	1	2.5	11.85^**^	16.0	52.84^**^
Habitat type (Ha)	1	0.7	3.13^ns^	0.2	0.61^ns^
Contaminant (Contam)	4				
C1—Control vs Carbaryl	1	0.1	0.49^ns^	0.0	0.00^ns^
C2—Control vs Iron	1	0 .0	0.42^ns^	0.0	0.00^ns^
C3—Control vs Metal	1	0.0	0.20^ns^	0.0	0.00^ns^
C4—Control vs Blank	1	0.2	0.91^ns^	0.0	0.00^ns^
Ha × Contam	4				
Ha × C1	1	1.3	5.28^*^	1.0	4.56^*^
Ha × C2	1	0.6	3.23^ns^	1.0	3.09^ns^
Ha × C3	1	0.8	3.01^ns^	1.3	4.15^ns^
Ha × C4	1	1.1	5.79^*^	0.2	0.82^ns^
Pooled	49	0.2		0.3	
Total	59				
Pair-wise tests		Not conclusive			

**Notes.**

ns, not significant (*p* > 0.05).

* *p* < 0.05.

** *p* < 0.01.

t = data were log(*x* + 1) transformed when Cochran’s test *p* < 0.05.

**Table 4:** Multivariate analyses of variance of the relative abundances (Bray-Curtis index) and composition (Jaccard index) of assemblages in each treatment. Site (Si) was random with 2 levels. Habitat type (Ha) and Contaminant (Contam) were fixed and orthogonal, with 2 and 5 levels, respectively; *n* = 3. Data were pooled when *p* > 0.25.

Source	df	Structure (Bray–Curtis index)		Composition (Jaccard index)	
		MS	Pseudo-*F*	MS	Pseudo-*F*
Site (Si)	1	21,240	9.37^**^	20,977	9.60^**^
Habitat type (Ha)	1	22,186	9.79^**^	8,081	3.70^**^
Contaminant (Contam)	4				
C1—Control vs Carbaryl	1	2,188	0.99^ns^	2,026	0.89^ns^
C2—Control vs Iron	1	2,575	1.17^ns^	1,722	0.76^ns^
C3—Control vs Metal	1	1,990	0.87^ns^	1,586	0.71^ns^
C4—Control vs Blank	1	2,435	1.16^ns^	1,822	0.78^ns^
Ha × Contam	4				
Ha × C1	1	3,807	1.72^ns^	4,125	1.82^*^
Ha × C2	1	3,198	1.45^ns^	2,890	1.27^ns^
Ha × C3	1	3,322	1.45^ns^	3,094	1.39^ns^
Ha × C4	1	3,425	1.62^ns^	2,199	0.94^ns^
Pooled	49	2,265		2,184	
Total	59				

**Notes.**

ns, not significant (*p* > 0.05).

* *p* < 0.05.

** *p* < 0.01.

Our results provide novel insight on the biotic responses to chemical disturbances. To increase our ability to predict effects of contamination on natural systems, it is crucial to incorporate information about species’ relationships with their habitats, considering possible synergistic effects of multiple stressors. Organisms are not equally susceptible to disturbance processes and their susceptibility is a function of not only the organism’s position in time and space relative to the disturbance, but also a function of the availability of substrate heterogeneities that act as refuges from the disturbance process (Woodin, 1978). Our results suggest that these relationships are context-specific and dependent, not only on habitat structure and the type of refugia that it provides, but also on the type of contaminant being released in the environment.

Sub-lethal concentrations of pesticides can cause important changes in behaviour such as foraging activity and refuge use, potentially having profound impacts on predator–prey interactions (Relyea & Edwards, 2010; Weis et al., 2001). Interactive effects of these contaminants and habitat structure can therefore mitigate or aggravate such impacts. A study on the effects of a pesticide and habitat structure on the behaviour and predation of a marine larval fish found that, although exposure to the contaminant increased the proportion of larvae with swimming abnormalities, prey mortality did not increase linearly with pesticide exposure. The authors found that mortality increased instead with habitat structure, suggesting that this could have been a consequence of compensating predator behaviour (Renick et al., 2015). Somewhat similar results were found in apple orchards, where an increase in the structural complexity of habitat influenced pesticide effects on predators (Lester, Thistlewood & Harmsen, 1998). In these systems, a pyrethroide pesticide killed great number of predators but not of prey; which could damage the crops. To maintain the number of predators, refuges were designed, increasing the complexity of habitat—which increased, to a certain point, the number of predators, mitigating the impacts of the pesticide (Lester, Thistlewood & Harmsen, 1998). Here, no mitigation effects of habitat structure were found. We found that carbaryl only reduced the number of species on more complex habitats, and this was probably mainly due to a reduction in the number of gastropods species. Interestingly, there were no clear effects of this pesticide on the total abundance of gastropods, indicating that the contaminant increased colonisation of particular species of this group at the complex habitats. We also found that the plaster itself affected the abundance of gastropods. An adverse effect of the plaster control on limpets was first identified from work on the limpet *Patella vulgata* (Cartwright, Coleman & Browne, 2006). There, the authors speculated that this could be as a result of the calcium oxide component of the plaster (Winder & Carmody, 2002) and simultaneously a local elevation in particulate matter as a result of the dispersion of the plaster within the water. It is interesting that this effect is general to the mollusc component of the assemblage but we can offer no explanation as to why. An interesting investigation to arise from this study would be to test the model that there is differential sensitivity to calcium oxide among aquatic invertebrates. Given the use of lime in building materials, this also a possible explanation for the paucity of mobile gastropods on seawalls and other artificial structures (Chapman, 2003).

The other two contaminants used here—iron phosphate and metaldehyde—are used to kill terrestrial gastropods such as slugs, acting on their salivary and epidermis glands (Moreau, Burgeot & Renault, 2015; Rae, Robertson & Wilson, 2009; Speiser & Kistler, 2002). Metaldehyde has also been shown to affect some individuals of the Pacific oyster (*Cassostrea gigas*; Moreau, Burgeot & Renault, 2015). Here, metaldehyde reduced the number of the species of gastropods, which was expected. As with carbaryl, however, the total abundance of gastropods was not affected, suggesting that the abundance of some species increased with the presence of this contaminant. Effects of iron phosphate were not as strong and no particular impacts on gastropods were observed.

One of the great challenges moving forward is how to incorporate evidence of biotic responses from individual disturbances to multiple stressors. The increased use of coastal habitats for recreational or economical activities, including trampling or collection of organisms living in intertidal habitats (Keough & Quinn, 1998; Thompson, Crowe & Hawkins, 2002), not only increases the likelihood of pollution, but also the loss and/or degradation of natural habitats (Crain et al., 2009). Predicting biotic responses to multiple stressors contaminants requires therefore a clear understanding of the complex ways these stressors might interact. Unravel these interactive effects is essential to underpin better design, planning and management strategies in ecological risk assessments.

## Supplemental Information

10.7717/peerj.1985/supp-1 Data S1 Raw data

## Additional Information and Declarations

### Field Study Permissions

The following information was supplied relating to field study approvals (i.e., approving body and any reference numbers): NSW Fisheries Permit Number: F96/146.

### Data Availability

The following information was supplied regarding data availability: The raw data has been supplied as Data S1.
